# Peer-teaching cardiac ultrasound among medical students: A real option

**DOI:** 10.1371/journal.pone.0212794

**Published:** 2019-03-27

**Authors:** Alon Ben-Sasson, Yotam Lior, Jonathan Krispel, Moshe Rucham, Noah Liel-Cohen, Lior Fuchs, Sergio L. Kobal

**Affiliations:** 1 Joyce and Irwing Goldman Medical School, Faculty of Health Sciences, Ben-Gurion University of the Negev, Beer-Sheva, Israel; 2 Soroka Clinical Research Center, Soroka University Medical Center, Beer-Sheva, Israel; 3 Cardiology Department, Soroka University Medical Center, Beer-Sheva, Israel; 4 Medical Intensive Care, Soroka University Medical Center, Beer-Sheva, Israel; King Saud University, SAUDI ARABIA

## Abstract

**Introduction:**

Teaching cardiac ultrasound (CU) image acquisition requires hands-on practice under qualified instructors supervision. We assessed the efficacy of teaching medical students by their previously trained classmates (teaching assistants [TAs]) compared to teaching by expert trainers (cardiologists or diagnostic medical sonographers.

**Methods:**

Sixty-six students received 8-hour CU training: 4-hour lectures on ultrasound anatomy and imaging techniques of 6 main CU views (parasternal long [PLAV] and short axis [PSAV]; apical 4-chamber [4ch], 2-chamber [2ch], and 3-chamber [3ch]; and sub costal [SC]) followed by 4 hours of hands-on exercise in groups of ≤5 students under direct supervision of a TA (**group A**: 44 students) or a qualified trainer (**group B**: 22 students). Students’ proficiency was evaluated on a 6-minute test in which they were required to demonstrate 32 predetermined anatomic landmarks spread across the 6 views and ranked on a 0–100 scale according to a predetermined key.

**Results:**

The 6-minute test final grade displayed superiority of group A over group B (54±17 vs. 39±21, respectively [p = 0.001]). This trend was continuous across all 6 main views: PLAV (69±18 vs. 54±23, respectively), PSAV (65±33 vs. 41±32, respectively), 4ch (57±19 vs. 43±26, respectively), 2ch (37±29 vs. 33±27, respectively), 3ch (48±23 vs. 35±25, respectively), and SC (36±27 vs. 24±28, respectively).

**Conclusions:**

Teaching medical students CU imaging acquisition by qualified classmates is feasible. Moreover, students instructors were superior to senior instructors when comparing their students' capabilities in a practical test. Replacing experienced instructors with TAs could help medical schools teach ultrasound techniques with minimal dependence on highly qualified trainers.

## Introduction

The cardiovascular physical examination is a cardinal part of the classical patient assessment process; however, its diagnostic yield is limited [1–3]. Therefore, physicians often rely on complementary studies for the diagnosis of cardiac diseases. The use of bedside cardiac ultrasound (CU) was shown to significantly improve cardiac diagnostic ability of cardiologists and non-cardiologists physicians [4,5]. Furthermore, increased diagnostic yield was also observed among medical students after brief training and was shown to surpass that of traditional physical examination based mainly on auscultation by board-certified cardiologists [6].

Past studies have substantiated that the integration of CU curriculum in medical-school is feasible [7–11] and that medical students are capable of using this modality to achieve accurate results [5,7,8,12,13]. With a growing base of evidence in support of its utility, the demand for integration of CU training into the medical-school curriculum is on the rise. Much like standard physical examination, CU imaging acquisition is traditionally taught in small groups by highly qualified instructors. However, shortage in qualified instructors continues to limit the incorporation of this important diagnostic modality to medical-school curriculums [14].

Previous research has shown that peer teaching is an effective way of introducing the principles of ultrasound examination to medical students in the fields of cardiac, abdominal, and musculoskeletal systems [6,7,15–20]. A focused estimation of student-teachers’ ability to teach practical CU skills to their peers is still needed in order to determine the effectiveness of this teaching method to large numbers of medical students.

In this study, we aim to prove that trained medical students are as capable as qualified instructors in teaching CU to their peers and to assess the efficacy of said teaching.

## Methods

### Study design and population

This was a prospective interventional study. The studied population comprised of medical students in their first clinical year from Ben-Gurion University of the Negev, Israel. All participating students were beginning a 3-month internal medicine rotation which curriculum included a mandatory basic cardiac ultrasound training. Students were informed that while their participation in the course was mandatory, participating in the study was on a voluntary basis and will not affect their final grade. The study was approved by the Soroka University Medical Center local ethics committee approval number SOR-14-0106. Written informed consent was obtained from the students.

### Ultrasound course

During the first 2 weeks of their Internal Medicine rotation, all students participated, for the first time in their medical education, in a mandatory introductory 8-hour echocardiography course. Details of the course and its efficiency are depicted in a previously published study by our group [9]. Briefly, teaching hours were divided into 4 hours of frontal lectures and an additional 4 hours of hands-on practice in the use of an ultrasound device. The *frontal lectures*, completed during the first week of the course, covered an introduction to ultrasound physics, and principles of 2-dimensional imaging and Doppler effect. Cardiac anatomy was reviewed from an echocardiography perspective and included the parasternal, apical, and subcostal views. Case studies with matching echocardiographic imaging were used in introducing the students to left ventricle function assessment, normal and pathologic valve structures, pericardial effusion, and inferior vena cava (IVC) width variations. *Hands-on* practice was performed in small groups of up to 5 students and was divided into two 2-hour sessions. In these sessions, students were taught the basic performance approach for a limited CU study and were instructed in the necessary techniques for acquisition of each view using 3 simple transducer maneuvers–correct alignment, rotation, and tilt. All practice examinations were performed on healthy willing student volunteers. Training was performed using standard US machines (Vivid S6, GE Healthcare, Buckinghamshire, UK) and portable ultrasound devices (PUD) (Vscan, GE Healthcare). The use of standard US machines fitted with larger screens provided a better teaching tool for large groups. The views practiced by the students were the parasternal long axis view (PLAV) and parasternal short axis view (PSAV) at 3 levels: base of the heart, mid-ventricle, and apex; the apical 4 (4ch), 2 (2ch), and 3 (3ch) chambers views, and the sub-costal (SC) view, with special regard to IVC changes with respiratory variation. After completion of the course, students were allowed access to PUD devices for self-practice on their peers or hospitalized patients after clarifying that this was a non-clinical, self-practice study and obtaining oral informed consent.

### The intervention

Prior to initiation of the *hands-on* portion of the course, students were randomly divided to 2 main groups by simple randomization over the students’ name list, in order to minimize selection bias. Group A was taught by 4 teaching assistants (TAs) and group B was taught by a cardiologist or a diagnostic medical sonographer. To minimize the effects of other factors, both groups were taught and trained simultaneously with the same devices in different teaching spaces and a constant ratio of trainees to trainers was maintained in each group. Staff availability limitations dictated that fewer students were allocated to group B in order to uphold these principles.

### Student teaching assistants

Four medical students in their third year of clinical education were assigned as teaching assistants by the PI. In addition to prior teaching experience, all TAs had previously completed the discussed introductory US course. An additional 8 hours of extra clinical and technical skills, methodology, and individual practice sessions were given by the PI to assure a similar standard of teaching between TAs.

### Qualified trainers

A board-certified cardiologist and a diagnostic medical sonographer participated in this study as qualified trainers. Both qualified trainers have been practicing echocardiography for many years and have previously taught POCUS to medical students and MD professionals in their internship and practice. Both have participated in this study on their free time on their own good will and personal interest in echocardiography teaching. A brief review of the views to be taught and teaching methodology was conducted by the PI to ascertain similar teaching standards, however no didactical preparation was included.

### Post-course evaluation

Two weeks after the last practice session, participating students were offered to fill out a demographic questionnaire (S2 Appendix) and undergo a 6-minute practical exam to assess their image-acquisition skills. This exam was used in previous studies by out group [7,9] and was not obligatory for the students. By agreeing to take the test, students approved their participation in this study.

Each student was evaluated individually. In order to standardize examination difficulty, all tests were performed on healthy models who were screened prior to the exam for good ultrasound viewing windows. The examination was overseen by a member of the teaching staff for timekeeping and PUD technical troubleshooting.

During the examination, each student was equipped with a PUD device and was given 6 minutes to demonstrate all taught cardiac views: PLAV, PSAV–base, mid-ventricle, and apex, Apical 4ch, 2ch, 3ch, and standard and IVC focused subcostal views. Students were instructed to record a 3-second video clip of each view once they believed an optimal image had been achieved.

To minimize possible analytical biases, several precautions were taken: (A) all data was collected using designated serial numbering without any identifying information; (B) all examination clips were analyzed by a cardiologist who did not participate in the teaching phase of the study, did not know the students, and was blinded to the designated group of the student; and (C) each echographic viewing window was graded on a 0–100 scale according to a predetermined checklist of anatomical landmarks wherein a complete demonstration of all landmarks was graded as 100 and inability to demonstrate any landmark was graded as 0 (S2 Appendix).

### Data analysis

The prefered analysis method for continous variables in this study was parametic using Student *t*-test. However, when parametric assumptions could not be satisfied, Mann-Whitney test was performed. Nominal variables were analyzed using Pearson’s χ2 test or Fisher exact test when needed. All the statistical tests were performed using 2 sided α = 0.05 with appropriate confidence intervals when needed. Data was analyzed using IBM SPSS statistics software. Post-hoc power calculations were performed to ascertain sufficient statistical power to reject the null hypothesis.

## Results

**Fig 1:**
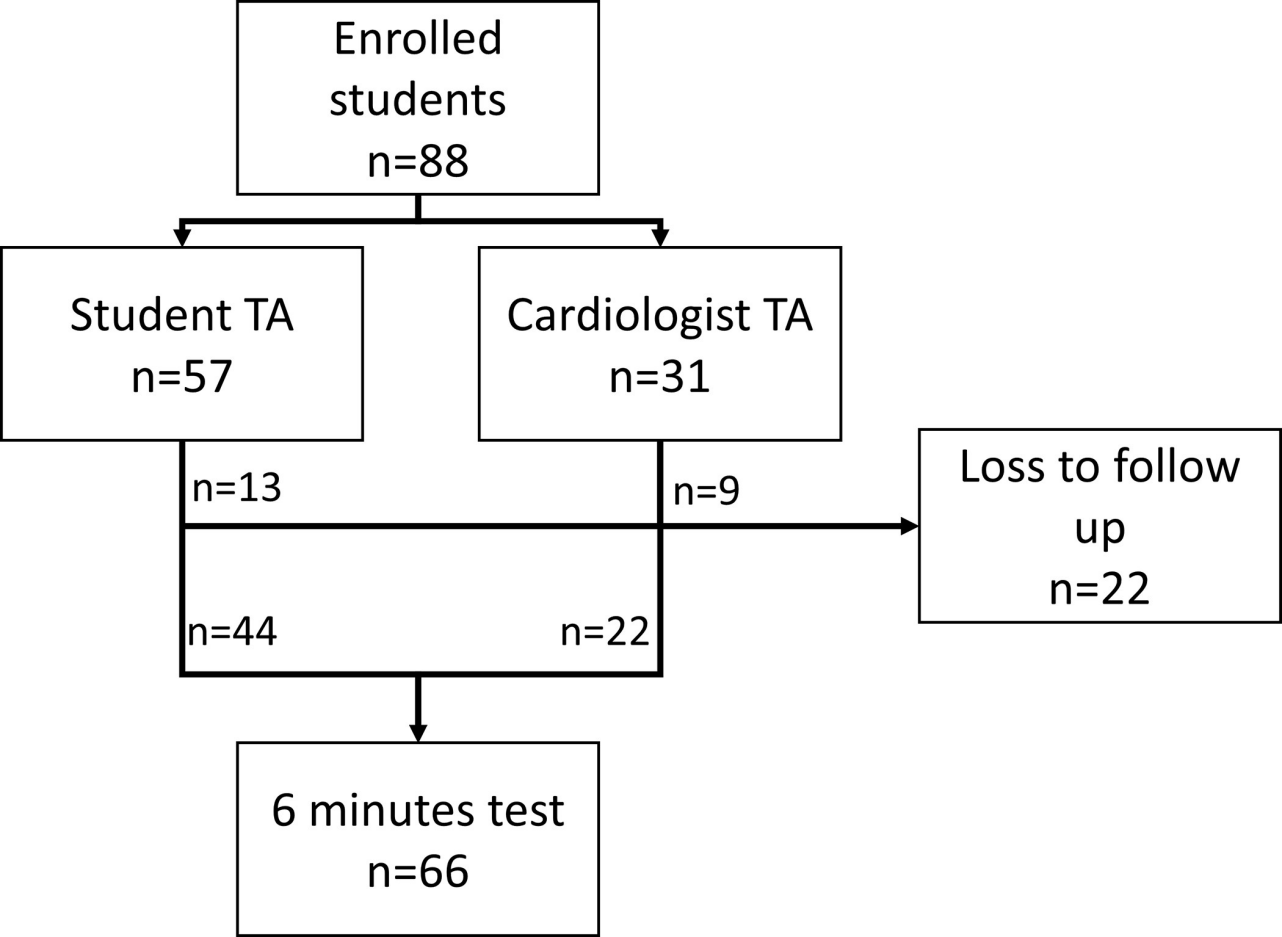
Cohort recruitment flowchart.

A total of 88 students were enrolled in the study and successfully completed the course. Twenty-two (25%) students (13 of the student TA group and 9 of the cardiologist TA group) did not attend the final 6-minute examination and were subsequently excluded from the study as lost to follow up, despite best efforts made by the research staff (**Fig 1**).

Table 1 depicts the demographic characteristics of the cohort. The TA taught group (group A) was composed of 44 students (males 63%) with mean age of 27±2 years. The qualified trainer taught group (group B) was composed of 22 students (males 50%), with mean age of 26±2 years. The 2 groups were comparable in all assessed variables.

**Table 1:** Cohort baseline characteristics.

Variable		Student TA Group A (n = 44)	Cardiologist TA Group B (n = 22)	*p*-value
Age, mean (SD), y		27 (2)	26 (2)	0.1
Gender, male, No. (%)		25 (63%)	11 (50%)	0.34
Work outside or med school, No. (%)		34 (77%)	13 (59%)	0.12
Former academic education, No. (%)		5 (11%)	2 (9%)	1
Reports home-practice of echocardiography, No. (%)		20 (45%)	8 (38%)	0.58
Past echocardiography experience, No. (%)		8 (18%)	4 (18%)	1
Level of interest in echocardiography, median (IQR)		4 (4–5)	4 (4–5)	0.67
Medical field of interest, No. (%)	Doesn’t know	14 (32%)	11 (50%)	n/a
	Internal	17 (39%)	5 (23%)	
	OBGYN	1 (2%)	3 (14%)	
	Pediatrics	4 (9%)	2 (9%)	
	Surgery	8 (18%)	1 (5%)	

The 6-minute test scores are detailed in **Table 2**. Group A achieved higher scores in 31 out of 32 elements assessed in the test, of which 14 were found to be statistically significant. Group A also attained a statistically significantly higher total examination score (54±17 vs. 39±21; *p* = 0.001).

**Table 2:** 6-Minute test scores.

Variable		Student TA Group A (n = 44)	Cardiologist TA Group B (n = 22)	*p*-value
Parasternal Long View, mean (SD)	Left Ventricle	64 (27)	52 (24)	0.1
	Mitral Valve	85 (23)	66 (36)	0.03
	Aortic Valve	68 (34)	55 (38)	0.15
	Right Ventricle	51 (20)	39 (26)	0.04
	Left Atrium	76 (25)	59 (33)	0.05
	Total Score	69 (18)	54 (23)	0.01
Parasternal Short View–Base, mean (SD)	Aortic Valve	43 (32)	25 (26)	0.03
	Tricuspid Valve	31 (27)	11 (21)	0.005
	Pulmonic Valve	17 (24)	5 (15)	0.03
	Right Ventricle	42 (19)	36 (23)	0.28
	Interatrial Septum	23 (25)	11 (21)	0.08
	Total Score	31 (18)	18 (14)	0.003
Parasternal Short View–Mid Ventricle, mean (SD)	Left Ventricle	58 (32)	41 (33)	0.05
	Mitral Valve	58 (34)	34 (36)	0.01
	Right Ventricle	35 (23)	25 (25)	0.11
	Total Score	50 (24)	33 (26)	0.01
Parasternal Short View–Apex, mean (SD)	Left Ventricle	59 (41)	34 (42)	0.03
	Papillary Muscles	39 (43)	27 (43)	0.25
	Total Score	49 (40)	31 (41)	0.06
Parasternal Short View Total, mean (SD)		65 (33)	41 (32)	0.006
Apical 4 Chamber View, mean (SD)	Left Ventricle	55 (18)	50 (27)	0.44
	Right Ventricle	49 (17)	39 (21)	0.04
	Mitral Valve	67 (28)	48 (36)	0.03
	Tricuspid Valve	65 (37)	43 (39)	0.03
	Interatrial Septum	48 (30)	36 (38)	0.16
	Total Score	57 (19)	43 (26)	0.06
Apical 2 Chamber View, mean (SD)	Left Ventricle	40 (28)	39 (26)	0.9
	Mitral Valve	43 (38)	34 (32)	0.39
	Right Atrium	27 (31)	25 (34)	0.7
	Total Score	37 (29)	33 (27)	0.51
Apical 3 Chamber View, mean (SD)	Left Ventricle	53 (27)	41 (29)	0.09
	Mitral Valve	65 (38)	43 (36)	0.03
	Aortic Valve	27 (29)	20 (25)	0.41
	Left Atrium	45 (24)	36 (28)	0.16
	Total Score	48 (23)	35 (25)	0.04
Apical View Total, mean (SD)				47 (17)	37 (22)	0.07
Subcostal Ventricle view, mean (SD)	Right Ventricle	49 (37)	36 (41)	0.19
	Interventricular Septum	32 (38)	25 (37)	0.43
	Interatrial Septum	16 (26)	16 (32)	0.69
	Total Score	32 (30)	26 (34)	0.23
Subcostal IVC view, mean (SD)	Morphology	55 (46)	32 (42)	0.06
	IVC Respiratory Variation	24 (25)	11 (21)	0.05
	Total Score	39 (36)	22 (31)	0.06
Subcostal View Total, mean (SD)				36 (27)	24 (28)	0.05
6-minute test total score, mean (SD)				54 (17)	39 (21)	0.001

### Parasternal views

*PLAV*–Group A attained higher scores in all 5 predetermined anatomical landmarks and achieved a higher total score (69±18 vs. 54±23; *p* = 0.01). *PSAV*–Group A attained higher scores than their peers in all assessed anatomical landmarks required for PSAV imaging and attained a higher total score (65±33 vs. 41±32; *p* = 0.006).

### Apical views

Group A achieved better visualization in all of the 12 anatomical sites assessed in the apical views. While group A was able to attain higher total scores in all apical views, the only statistically significant result was found to be in the 3-chamber view total score (48±23 vs. 35±25; *p* = 0.04).

### Subcostal view

In this view as well, group A achieved a significantly higher overall score (36±27 vs. 24±28; *p* = 0.05). While the sole statistically significant finding between the two groups was in the acquisition of the IVC respiratory variation (24±25 vs. 11±21; *p* = 0.05), group A students managed to achieve higher or identical grades in the rest of the 4 landmarks, albeit without any statistical significance.

## Discussion

In this study, we have shown that a short hands-on CU course, led by medical students as teaching assistants, provided novice participants the ability to perform basic cardiac ultrasound studies. While these results are similar to results presented in previous studies such as those of Gradl-Dietsch et al. [20], when compared to the group taught by board certified cardiologists and diagnostic medical sonographers, students taught by their peers demonstrated better image-acquisition ability in 31 out of the 32 predetermined anatomical landmarks evaluated in this study, 14 of which were statistically significant.

These results are somewhat unexpected considering that each TA student was practically a novice himself with approximately 15–20 hours of echocardiography experience prior to their first teaching session. However, it is possible that this detail is the reason for our student TAs’ success in teaching. Being novices themselves, they did not suffer from the cognitive bias named "the curse of knowledge"–a state in which an experienced individual is unable to share his or her knowledge with a novice listener due to discrepancy between their perceptions of the same subject [21]. Being closer to their student peers in their experience and understanding of echocardiography, student TAs were able to provide a more focused approach to teaching and to quickly troubleshoot any difficulties their students had. Another possibility one has to consider is that students in this study were evaluated on their ability to demonstrate and capture images of normal anatomy by ultrasound. It is not unthinkable that had students been evaluated for their ability to acquire and interpret pathological images or otherwise apply advanced CU techniques, TAs’ teaching abilities may have reached their limits, as shown in other studies [22]. While our data strongly supports TAs’ ability to teach cardiac sonography, the limits of this ability need to be explored in future studies.

The benefits of peer-teaching in medical education are well established [23–26]. Previous US imaging studies have shown peer-teaching to be promising in this field but were limited by small heterogenic cohorts [16–18]. To the best of our knowledge, our work was conducted using the largest cohort in this type of study to date. Our group's results differ from the work of Kühl et al. [18], who compared the teaching capabilities of focused emergency echocardiography between student TAs and cardiologists, with OSCE results supporting the latter group. While innovative, Kühl assessed only two views (five-chamber and PSLX) and studied a small cohort of 30 students in various years of their medical education. We believe that given a larger and more homogenous cohort as well as more examination views, the results of Kühl et al. might have tilted in favor of the TA group as well.

Previous works in this field of research have used a blend of cardiac, abdominal, and musculoskeletal US imaging as their goal. Our study focused solely on the acquisition of CU imaging, traditionally regarded as the hardest to obtain. Students trained by their peers were superior to students trained by qualified instructors also in this adverse field of ultrasound.

Although students taught by TAs reached an overall higher score compared with students taught by qualified trainers, it is evident that both groups have reached sub-optimal competence in the final examination (54±17 vs. 39±21 in the TAs and qualified trainers, respectively). These results could be explained by either didactical difficulties, disparity between the expected level of competence in the exam and the realistic level to be expected from novice practitioners after a short course or even unfit examination conditions. Future studies should try and fit students with a more tailored scoring system and optimal testing conditions to better reflect students’ abilities.

It is evident that both groups performed better at demonstrating parasternal views compared with apical views. These findings could be attributed to the comfortable anatomical position of the parasternal views, with more prominent anatomical markers present in this area and less chance of probe slippage when good technique is used. Another factor that might influence these results may be that the apical views were the last to be obtained by the students with consequent fatigue in the transducer-holding hand, a very common phenomenon among novice US practitioners.

As the demand for CU studies is on constant rise in clinical applications and patient management, the demand for healthcare providers qualified in this field is expected to rise as well. Focusing the attention and time of experienced professionals to complicated cases is thought to increase their relative impact. However, achieving this requires that more healthcare providers will be able to perform and interpret basic CU as an augmentation study to their physical examination. Although the number of medical schools already implementing ultrasound training in their curriculum has expanded dramatically in recent years, it has yet to conquer the majority of them.

Data collected in the works of Bahner et al. [27] and Dinh et al. [28] has shown that the integration of ultrasound teaching in the curriculum of medical schools in the United States is highly variable in duration as well as stage of medical training (e.g., pre-clinical or clinical years). In both works it is evident that the main roadblocks which inhibit further integration of ultrasound teaching are lack of funds, skilled instructors and adequate workspace. It is logical to assume that as more and more medical schools choose to implement CU teachings, the demand for qualified CU instructors will increase accordingly.

Our study establishes that the use of peer-teaching is a feasible solution for the shortage of available expert CU instructors and may reduce teaching-associated costs. More research is needed regarding long-range knowledge retention of CU skill and students’ views of this method of teaching.

Our study suffered from a few limitations. First, in order to maintain similar examination conditions, all CU examinations were performed on young healthy models that were known to have good acoustic windows and not real-life medical patients. Second, while all participating TAs were chosen based on their technical and didactical skills, qualified trainers participated in the course based on their availability and willingness. It is possible that some of the results could be explained by didactical variances between the groups. Therefore, we believe that future courses should include a pre-course didactical workshop to ascertain similar teaching abilities between instructors. Third, we used a single blinded rater to assess students’ performance in the six-minute examination. We believe that adding another rater to the study could have improve the credibility of the results. Fourth, in this study we have implemented a 6-minute examination to assess students’ performance. It is possible that students’ performance could have been better had more time been given for the examination, as in many other academic fields. Additionally, it is possible that under a different assessment methodology, such as those mentioned by Gaudet et at. [29], students’ performance could have been altered. Fifth, our study is limited in its generalizability given that it was performed in a single center with a single medical school class. Sixth, the study was not designed to assess long-term skill retention among students since the 6-minute test was held 2 weeks after the course. Seventh, the number of students that did not attend the 6-minutes test was significantly high. Probably most of the students that preferred not to be examined were those that felt insecure on their own ultrasound skills. It is possible that the results would be different if these students were included in the final analysis. It is important to note, however, that the number of students removed from final analysis was similar between groups (13 students from the TA group and 9 students from the qualified instructors group). Finally, both groups had students who were previously exposed to echocardiography to some extent prior to their participation in our course ranging from pure observance of a physician performing an exam to a brief trial of using a device. Since the exposed students composed an identical portion in both groups (18%), they were included in the study.

## Conclusions

Teaching cardiac ultrasound to medical students by their classmates is feasible and effective. Furthermore, students taught by their classmates displayed superior CU acquisition skills compared to counterparts trained by experienced tutors. The use of students as mentors may be a practical strategy to overcome some of the impediments existing in integration of ultrasound technique teaching to medical school curriculum.

## Supporting information

S1 Appendix Student demographic questionnaire. (DOCX)

S2 Appendix 6-minute echocardiography test scoring system. (DOCX)

S1 Table Raw data and 6 minute test score standardization. (XLSX)
