# Supplementary material for: Peer-teaching cardiac ultrasound among medical students: A real option
Source: PLoS One. 2019 Mar 27;14(3):e0212794. doi: 10.1371/journal.pone.0212794 (PMC6436682; doi:10.1371/journal.pone.0212794)
Supplement: S1 Appendix — (DOCX) [file pone.0212794.s001.docx]

**S1 Appendix – Student demographic questionnaire**

**Demographic questionnaire – Basic echocardiography course**

**Sex:** 1. Male 2. Female

**Age:** _______________

**Familial status:** 1. Single 2. Married 3. Other

**Number of children**: ___

**Country of origin:** 1. Israel 2. Other **Year of immigration to Israel:** _____

**Military service:** 1. Yes 2. No **National Service:** 1. Yes 2. No

**Area of residence in Israel (originally):** 1. South 2. Center 3. North 4. Jerusalem

**Employment during medical school:** 1. Yes 2. No

**Previous academic degrees:**
1. Yes 2. No Field of research/studies: _______________

**Previous technical background (Mechanics, Carpentry etc.):**
1. Yes _________ 2. No

**How would you rate your ability to "Work with your hands"?**
1. Above average 2. Reasonable 3. Below average

**Did you have any experience with echocardiography (as a patient or out of interest)?**
1. Yes 2. No

**Rate your interest level in learning how to use an echocardiography device (1=very low, 5=very high):**
1 2 3 4 5

**At this point in your studies, which field of medicine is most appealing to you?**
