# Supplementary material for: Peer-teaching cardiac ultrasound among medical students: A real option
Source: PLoS One. 2019 Mar 27;14(3):e0212794. doi: 10.1371/journal.pone.0212794 (PMC6436682; doi:10.1371/journal.pone.0212794)
Supplement: S2 Appendix — (DOCX) [file pone.0212794.s002.docx]

**Appendix B – 6-minute echocardiography test scoring system**

**Participant Serial Number:_______**

| **PARASTERNAL LONG AXIS VIEW** | | | |
| --- | --- | --- | --- |
| 0 Incorrect | 1 Correct alignment (horizontal heart) | | **LEFT VENTRICLE** |
| 0 Partial LV endocardial demarcation | 1 Total LV endocardial demarcation | |  |
| 0 Not visualized | 1 Anterior leaflets excursion | | **MITRAL VALVE** |
| 0 Not visualized | 1 Posterior leaflet excursion | |  |
| 0 Not visualized | 1 Partial cusps excursion | 2 Total cusps excursion | **AORTIC VALVE** |
| 0 Not visualized | 1 Partial visualization | 2 Endocardial visualization | **RIGHT VENTRICLE** |
| 0 Not visualized | 1 Partially exposed | 2 Completely exposed | **LEFT ATRIUM** |
| __ of 10 possible points | | | **TOTAL SCORE** |


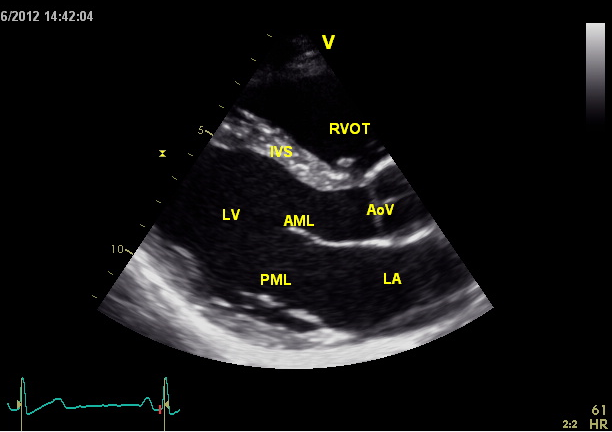


| **PARASTERNAL SHORT AXIS VIEW** | | | | |
| --- | --- | --- | --- | --- |
| 0 Not visualized | 1 Partial visualization | 2 Complete visualization | **AORTIC VALVE (leaflet number)** | **BASE** |
| 0 Not visualized | | 1 Visualization | **TRICUSPID VALVE** |  |
| 0 Not visualized | | 1 Visualized | **PULMONIC VALVE** |  |
| 0 Not visualized | | 1 Visualized | **RIGHT VENTRICLE** |  |
| 0 Not visualized | | 1 Visualized | **INTERATRIAL SEPTUM** |  |
| __ of 6 possible points | | | **TOTAL SCORE** | |
| **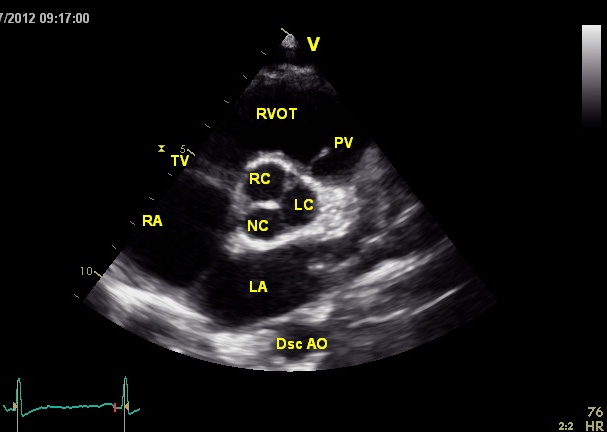** | | | | |
| 0 Not visualized | 1 Partial visualization | 2 Complete endocardial visualization | **LEFT VENTRICLE** | **MID-VENTRICLE** |
| 0 Not visualized | | 1 Anterior leaflet visualization | **MITRAL VALVE** |  |
| 0 Not visualized | | 1 Posterior leaflet visualization |  |  |
| 0 Not visualized | | 1 Visualization | **RIGHT VENTRICLE** |  |
| __ of 5 possible points | | | **TOTAL SCORE** | |
| 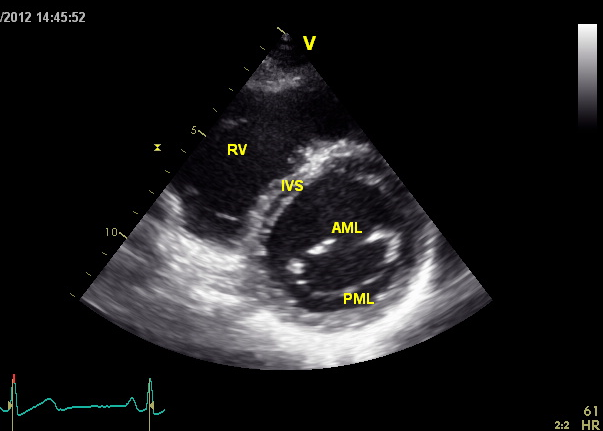 | | | | |
| 0 Not visualized | 1 Partial visualization | 2 Complete endocardial visualization | **LEFT VENTRICLE** | **APEX** |
| 0 Not visualized | | 1 Antero-lateral visualization | **PAPILLARY MUSCLES** |  |
| 0 Not visualized | | 1 Postero-medial visualization |  |  |
| 0 Not visualized | | 1 Visualization | **RIGHT VENTRICLE** |  |
| __ of 5 possible points | | | **TOTAL SCORE** | |
| 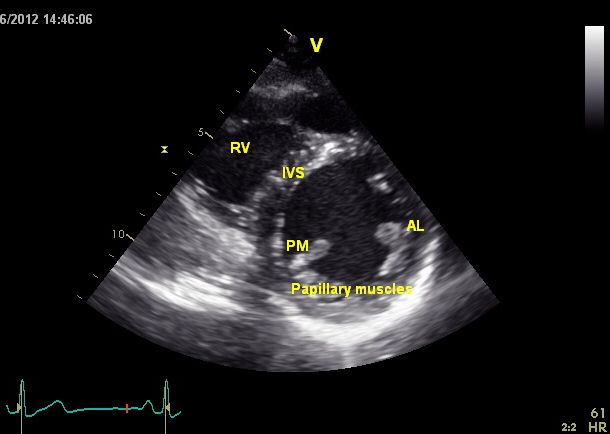 | | | | |

| **APICAL 4 CHAMBER VIEW** | | | |
| --- | --- | --- | --- |
| 0 Not visualized | 1 Partial visualization | 2 Complete endocardial visualization | **LEFT VENTRICLE (6 segments)** |
| 0 Not visualized | 1 Partial visualization | 2 Complete endocardial visualization | **RIGHT VENTRICLE (free wall)** |
| 0 Not visualized | 1 Partial leaflets excursion | 2 Complete leaflets excursion | **MITRAL VALVE ANATOMY** |
| 0 Not visualized | 1 Partial leaflets excursion | 2 Complete leaflets excursion | **TRICUSPID VALVE ANATOMY** |
| 0 Not visualized | 1 Partially exposed | 2 Completely exposed | **INTERATRIAL SEPTUM** |
| __ of 10 possible points | | **TOTAL SCORE** | |
| 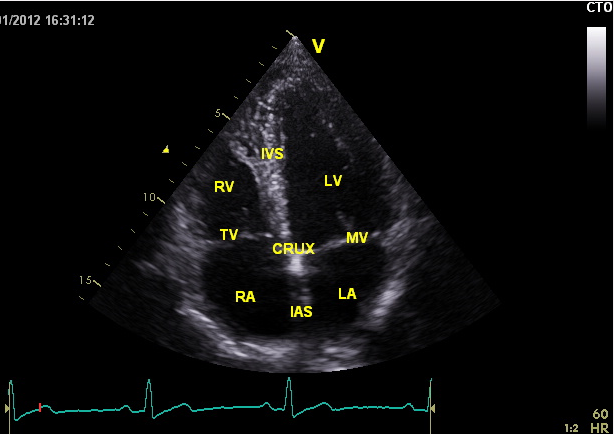 | | | |

| **APICAL 2 CHAMBER VIEW** | | | |
| --- | --- | --- | --- |
| 0 Not visualized | 1 Partial visualization | 2 Complete endocardial visualization | **LEFT VENTRICLE (6 segments)** |
| 0 Not visualized | 1 Partial leaflets excursion | 2 Complete leaflets excursion | **MITRAL VALVE ANATOMY** |
| 0 Not visualized | 1 Partially exposed | 2 Completely exposed | **LEFT ATRIUM** |
| __ of 6 possible points | | **TOTAL SCORE** | |
| 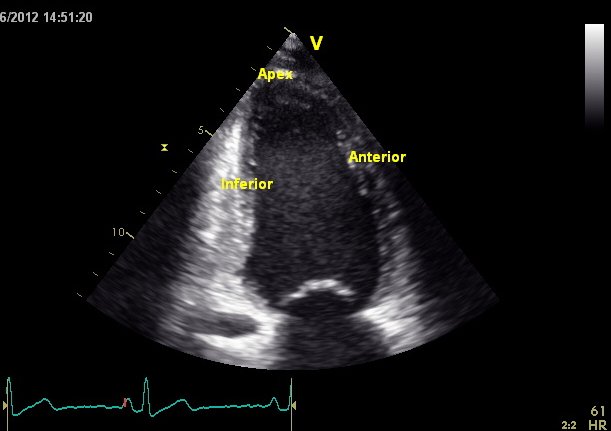 | | | |

| **APICAL 3 CHAMBER VIEW** | | | |
| --- | --- | --- | --- |
| 0 Not visualized | 1 Partial visualization | 2 Complete endocardial visualization | **LEFT VENTRICLE** |
| 0 Not visualized | 1 Partial leaflets excursion | 2 Complete leaflets excursion | **MITRAL VALVE ANATOMY** |
| 0 Not visualized | 1 Partial cusps excursion | 2 Total cusps excursion | **AORTIC VALVE** |
| 0 Not visualized | 1 Partially exposed | 2 Completely exposed | **LEFT ATRIUM** |
| __ of 8 possible points | | **TOTAL SCORE** | |
| 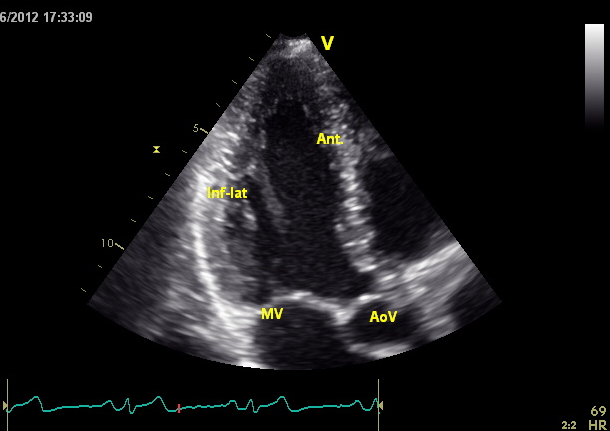 | | | |

| **SUBCOSTAL VIEW** | | | | |
| --- | --- | --- | --- | --- |
| 0 Not visualized | 1 Partial visualization | 2 Complete endocardial visualization | **RIGHT VENTRICLE (free wall & apex)** | **VENTRICLES** |
| 0 Not visualized | 1 Partially exposed | 2 Completely exposed | **INTERVENTRICULAR SEPTUM** |  |
| 0 Not visualized | 1 Partially exposed | 2 Completely exposed | **INTERATRIAL SEPTUM** |  |
| 0 Not visualized | 1 Partially exposed | 2 Completely exposed | **MORPHOLOGY** | **INFERIOR VENA CAVA** |
| 0 Unable to assess | | 1 Able to assess | **RESPIRATORY VARIATION** |  |
| __ of 9 possible points | | | **TOTAL SCORE** | |
| 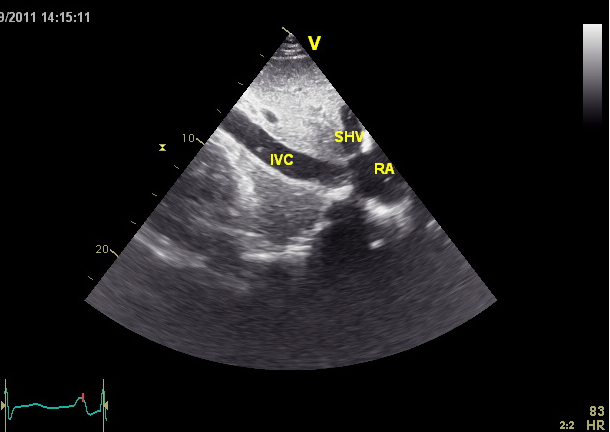 | | | | |
